# Proposal for Manual Osteopathic Treatment of the Phrenic Nerve

**DOI:** 10.7759/cureus.58012

**Published:** 2024-04-11

**Authors:** Bruno Bordoni, Allan R Escher, Maciej Duczyński

**Affiliations:** 1 Physical Medicine and Rehabilitation, Don Carlo Gnocchi Foundation, Milan, ITA; 2 Anesthesiology/Pain Medicine, H. Lee Moffitt Cancer Center and Research Institute, Tampa, USA; 3 Osteopathy, Triada Zdrowia Clinic, Warsaw, POL

**Keywords:** myofascial, pain, phrenic nerve, diaphragm, osteopathic manipulation, osteopathy, fascia

## Abstract

The article reviews the anatomical path of the phrenic nerve and its anastomoses, with the most up-to-date knowledge reported in the literature. We have briefly reviewed the possible phrenic dysfunctions, with the final aim of presenting an osteopathic manual approach for the treatment of the most superficial portion of the nerve, using a gentle technique. The approach we propose is, therefore, a theory based on clinical experience and the rationale that we can extrapolate from the literature. We hope that the article will be a stimulus for further experimental investigations using the technique illustrated in the article. To the authors' knowledge, this is the first article that takes into consideration the hypothesis of an osteopathic treatment with gentle techniques for the phrenic nerve.

## Introduction and background

The peripheral nervous tissue that exits the spinal cord, constituting the spinal nerve, is organized into nerve fiber bundles; the latter are multiple axons protected by connective tissue or meningeal layers [1]. There are four connective layers. The circumneurium or paraneurium is the most external connective layer, which delimits the subparaneurial compartment; this tissue appears to be acellular with variable thickness [2,3]. This layer contacts the collagen of the adventitia of the vessels and the epimysium of the neighboring muscles; the space between the circumneurium and the collagen of the other structures is filled by adipose tissue [4]. Below the circumneurium, we can find the epineurium, with mechanical shock absorber functions, which is rich in collagen fibers [4].

This layer (and so all the other layers) is nourished by blood vessels (vasa nervorum) with a predominantly oblique and slightly coiled direction, like the lymphatic vessels, so that the vessel must not be disturbed by the movement of the nerve and protect the tissue from possible ischemia [5]. The epineurium is innervated by complex mechanoreceptor/nociceptor/nocifensive fibers (nervi nervorum), as are the underlying layers [5]. Below the epineurium, there is an internal or deep epineurium, which is supported by loose connective tissue and adipose tissue, which separates the perineurium [4]. Internal epineurium separates individual fascicles, allowing correct sliding of the fascicles; the epineurium with its external or superficial and semi-permeable side covers the entire nerve. Blood vessels are in the epineurium [4,5].

The collagen of the perineurium covers multiple fascicles and encounters the endoneurium. The perineurium presents a strong tension to protect the fascicles from excessive mechanical stress [6]. The endoneurium surrounds a single nervous fascicle, encountering the neurilemma/myelin sheath; the vessels penetrate the endoneurium [7]. One of the functions of this layer is to protect the nerve's ability to send electrical signals. Schwann cells produce collagen for the endoneurium in a greater percentage than the fibroblasts present, the latter of which are present in low quantities [8]. Between the nerve cells and the internal portion of the endoneurium, there is the endoneurial fluid, which is mainly cerebrospinal fluid, 70% water, with poor protein components; the fluid is non-compressible (hydrostatic pressure of approximately 2-3 mmHg) [8]. Endoneurial fluids are transported via the vascular wall and a mechanism called convective proximo-distal endoneurial fluid flow (movement of 3-8 millimeters per hour) [8].

The nervous branch that branches off from the neural body or axon (protected by the meningeal layers) can have ramifications that can encounter other nervous structures (neural somata, dendrites, axons), or other non-neural tissues, i.e., the telodendron [9]. The telodendron allows the creation of a more complex and articulated communication network, bringing multiple structures into contact and improving tissue functions [9]. The dendrite is a minor branch of the neural soma, which receives information specifically returning to the soma (centripetal direction) [9].

In the peripheral nervous system, neurolemmocytes or Schwann cells with their plasmalemma wrap the axon discontinuously, forming myelin (electrical insulator); the non-continuity of myelin allows the presence of Ranvier nodes or myelin sheath gaps. This anatomical construction allows for faster propagation of the electrical impulse [9]. An axon with a greater volume (axonal size and quantity of myelin) has a greater electrical activity and impulse velocity, compared to an axon with a smaller diameter [9].

The osteopathic medicine (OM) approach involves manual treatment of the peripheral and cranial nerves and autonomic systems directly and indirectly. A recent article demonstrated a change in the response of the vagus nerve in patients with epilepsy, working the area of parasympathetic abdominal innervation (in indirect mode and not with the hands on the nerve) [10]. OM to release mechanical tensions at the level of the occipitomastoid suture, where the jugular foramen and the exit of the vagus nerve (and glossopharyngeal and accessory nerves) are present, allows to improve the systemic parasympathetic response [11].

A manual therapy study demonstrated that it is feasible to palpate the median, radial, and ulnar nerve to test for hyperalgesia in patients with hand osteoarthritis [12]. Nerve palpation is feasible and useful to identify and measure (with an algometer) the mechanosensitivity of neural tissue, not only for the nerves of the upper limb but also for the nerves of the lower limb (femoral, common peroneal, tibial, sciatic nerves) [13]. Some regions of the body, following the anatomy of the nerve path, offer the possibility of palpating some more superficial portions, through light pressure starting from the skin. The manual approach to the nervous tissue aims to improve the function of the nerve after trauma [14].

The article reviews the anatomical path of the phrenic nerve or phrenicus nerve, and the rationale for being able to manually work a small superficial portion of the nerve. To the authors' knowledge, this is the first article to discuss the OM hypothesis for the phrenic nerve.

## Review

Path of the phrenic nerve: cervical area

The phrenic nerve is complex, with efferent and afferent pathways (the latter represent 30-45% of the total). It includes motor fibers (myelinated cholinergic), myelinated sensory fibers of large diameter (group Ia, Ib, II) and small diameter (group III), non-myelinated peptidergic type IV fibers, and non-myelinated adrenergic fibers of the orthosympathetic type (coming from the ganglia cervical sympathetic nerves) [15,16]. Of the axons, 60% are myelinated, while the remainder are unmyelinated [16].

Phrenic motor neurons originate from lamina IX of the spinal cord, the medial portion of the ventral horn [17,18]. They exit as nerve fibers from the ventral cervical area C3-C5 in most individuals; the phrenic nerve can exit, in rare cases, only from the C3-C4 area [19]. On exit, the phrenic nerve anastomoses with the ansa cervicalis, putting itself in direct communication with the cervical plexus (C1-C4) and with the vagus nerve (X) [20]. Indirectly, these connections suggest that the phrenic nerve receives or sends information to the muscles innervated by the ansa cervicalis: sternomastoid, trapezoid, sternohyoid, sternothyroid, omohyoid, thyrohyoid, geniohyoid, and vocal cords [21,22]. Indirectly, through the anastomosis with the X, cranially and extracranially, it contacts the glossopharyngeal nerve (IX) [23]. Indirectly, the root of C1, C2, and C3 connects the accessory nerve (XI) with the phrenic; in some cases, the anastomosis is directed via the ansa cervicalis [24,25]. The ansa cervicalis connects the phrenic nerve with the hypoglossal nerve (XII) and the supraclavicular nerve [24].

In its course, the phrenic nerve has communicating branches, which connect the entire brachial plexus, with the suprascapular, subclavian, dorsal scapular nerve, lateral pectoral, axillary, musculocutaneous, and radial nerve [26,27]. Before entering the mediastinum, the phrenic nerve anastomoses with the stellate ganglion or cervicothoracic ganglion, often forming plexiform structures [27,28]. The phrenic nerve can anastomose with the subclavian ansa, the nerve that communicates directly with the stellate ganglion and the middle sympathetic ganglion, and the laryngeal recurrent nerve of the vagus [28]. The subclavian ansa forms a ring with the subclavian artery (in front or behind the artery), and with different variations between right and left individuals, it links the middle and lower cervical ganglion [28]. Indirectly, via the stellate ganglion, the phrenic nerve can be put in communication with the visceral sympathetic system, such as the nerves to the larynx, trachea, pharynx, and esophagus, and inferior cardiac nerves [29]. The sympathetic component serves the phrenic to regulate the blood flow to the diaphragm muscle, through variation in the caliber of the blood ducts [19].

The phrenic nerve can have many accessory pathways, affecting between 60% and 85% of individuals [19,30]. The path of the phrenic accessory nerves of the cervical tract, deriving mainly from the ansa cervicalis, generally passes in front of the subclavian vein to cross the mediastinum [30]. Subdiaphragmatic accessory phrenic nerves may exist, but we have little data [31].

The phrenic nerve rests on the anterior scalene muscle fascia, anteriorly and in front of the prevertebral fascia; it passes behind the fascia of the omohyoid muscle (where the transverse cervical and transverse scapular vessels pass), and laterally-posteriorly to the clavicular muscular area of the sternocleidomastoid muscle (laterally the head of the clavicle) or Erb's point, near the cricoid cartilage [20,32,33]. The phrenic nerve bilaterally enters the mediastinum between the vein and the subclavian artery [20]. The thoracic duct crosses the left phrenic nerve anteriorly [34].

Mediastinal area

When the phrenic nerve enters the mediastinum, it crosses the internal mammary artery or internal thoracic artery near its origin (subclavian artery) [35,36]. The phrenic nerves enter the mediastinum together with the phrenic veins and arteries, constituting the pericardiacophrenic neurovascular bundle, passing in front of the pulmonary roots (above the middle of the mediastinal area), where major bronchi and the pulmonary arteries and veins are present [37,38]. The phrenic nerves contact the parietal pleura and pericardium.

The left phrenic nerve passes in front of the left vagus nerve lateral to the aortic arch, and over the pericardial area of the left ventricle (touching the left parietal pleura); compared to the right nerve, it is longer, as it must go beyond the ventricle and the diaphragmatic dome is lower than the right dome [39]. Before reaching the diaphragm, the nerve has a small “free” portion of approximately 72.5 millimeters [40].

The right phrenic nerve is deeper and shorter, with a more vertical direction; it travels laterally to the superior vena cava and right innominate vein, rests on the pericardial area of the right atrium, and contacts the right parietal pleura [39]. We can find a "free" portion of the nerve, before fusing into the diaphragm muscle, of approximately 60 millimeters [40].

The phrenic nerves provide small branches to the pericardial sac near the root of the lungs (pericardial branch), and the parietal pleura (parietal branch) [41,42]. The conduction velocity of the right phrenic nerve is faster than the left (6.94 meters per second on the right, 6.61 meters per second on the left), for a different length [43].

The pericardium (and some myocardial fibers) merges with a small portion of the superior and inferior vena cava (above and below the diaphragm); the pericardial/myocardial fibers appear to be innervated by the right phrenic nerve, which has catecholaminergic characteristics [44,45].

Subdiaphragmatic and abdominal area

The phrenic nerves innervate the diaphragm muscle, but do not end their journey in the muscle; the latter continue arriving in the abdomen. Past the diaphragm, the nerves or phrenicoabdominal branches form several subdiaphragmatic phrenic ganglia (especially on the left), anastomose with the celiac ganglion, the superior mesenteric ganglion and the aorticorenal ganglion, to end in the suprarenal gland; the phrenic nerves form a plexiform neural network with the sympathetic system and part of the abdominal vagus nerve [46-48]. The right phrenic nerve crosses the diaphragm from the foramen caval or in a smaller percentage, piercing the connective area of the muscle or phrenic center. The exit of the left nerve occurs mainly behind the central phrenic tendon or lateral to the esophageal hiatus [48]. Probably, the phrenic ganglia and their connections under the diaphragmatic surface could have a sympathetic vasomotor role, with an increase in blood pressure [19,49]. The phrenic nerves innervate the parietal peritoneum, Glisson's capsule, the liver (crossing the hilus), the gallbladder, the greater curvature of the stomach, falciform, and coronary ligaments of the liver [27,50-53].

The peripheral nerve can be palpated

An inflamed nerve (edema within the different fascial layers) becomes hypersensitive to mechanical stimulation, such as compression and traction, although the conduction of the nerve impulse may not change [54]. Every time the body is subjected to movement, the nerve of a limb or trunk must adapt to the same movement; if the nerve increases its diameter (presence of edema), or the tissue it passes through is not compliant during bodily actions, the symptom may arise and stimulate the production of ectopic electrical activity [54]. Symptoms can vary and do not always appear simultaneously or only with some movements, such as pain, paresthesia, and phenomena of allodynia and sensitization [55].

There are various provocative, neurological, and orthopedic tests, as well as the lengthening of the limb by an operator (neurodynamic tests) to try to identify the presence of dysfunctions of the peripheral nervous pathways [54]. Palpation of a peripheral nerve in its most superficial region is feasible and useful to verify tolerance to mechanical pressure, with moderate to substantial reliability [12,13,54-57].

Palpation to elicit a dysfunctional response of the nervous tissue, in the presence of inflammation, is also feasible for some portions of the cranial nerves [58].

Rationale for osteopathic treatment of the phrenic nerve

OM bases its manual approach on various techniques, including gentle "listening" techniques, where the clinician does not induce any movement [59]. The operator places his fingers or hand on the tissue chosen for the treatment; once the tissue is contacted, the pressure does not increase, and the perceived tissue tension does not change [59]. Imagine placing your fingers on a rose petal; no pressure is needed to evaluate the consistency of the flower. The listening technique ends when the tension of the worked tissue changes positively: the tension is reduced or the movement expressed by the palpated tissue is homogeneous [58,59].

The literature is extensive in describing local and systemic benefits in response to gentle manual treatments. We do not always understand in detail the mechanisms that allow positive adaptations to occur with the gentle manual approach. We know that the systemic and local inflammatory response decreases, the pain perceived (chronic or acute) by the patient decreases significantly, tissue microcirculation improves, viscoelastic properties improve, and systemic parasympathetic activity increases [60-67]. The manual approach involves all tissues and body systems, with a probable starting point from a more physiological metabolic behavior of the fibroblasts; the latter improves tissue tone and metabolism, with a positive effect throughout the body [68-70].

Recall that the meningeal layers covering the nerve are enclosed in the classification of fascial tissue, which is rich in fibroblasts [71]. By improving the behavior of fibroblasts, it increases the relationship between the pressures of the extracellular fluids and the sliding capacity between the different tissues (the viscoelastic properties improve); this mechanism avoids the formation of adhesions and further inflammatory processes [72,73]. Furthermore, they improve the electrical environment of the tissue/tissues of which they are part or with which they come into contact [4,72,73].

The physiological sliding of the tissues allows an optimal piezoelectric response, that is, a balanced transfer of electrons between the different tissue components (from DNA to collagen), thanks to the conversion of the mechanical energy produced by the movement into electrical polarization. As regards the nerve, this mechanism, called galvanotaxis or electrotaxis, allows the transport of multiple substances (growth factors, stem cells, etc.) to keep the meningeal tissue healthy [74].

The gentle osteopathic manual approach has no side effects, both in the presence of chronic pathologies and in the presence of patients who have undergone median sternotomy for a few days [75,76].

Phrenic dysfunctions

There are many causes that can damage the function of the phrenic nerve, partially or totally. The presence of spondylosis can cause foraminal stenoses (attributable to osteophyte), with transient paresis of the phrenic nerve [77]. A surgical approach to eliminate a neck malignancy could injure the phrenic nerve, although uncommonly; in rarer cases, removal of the cavoatrial tumor thrombus could damage the right phrenic nerve [78,79].

Neurological pathologies can damage the phrenic, such as Charcot-Marie-Tooth, with non-recoverable diaphragmatic paresis, as well as metabolic pathologies (diabetes), or due to viral causes (herpes zoster) [80-82].

Other visceral, cardiopulmonary, such as chronic heart failure or chronic obstructive pulmonary disease, and rheumatological (fibromyalgia) pathologies can damage the phrenic pathways, causing permanent dysfunction of the diaphragm [83-85]. Iatrogenic damage to the nerve can occur after traumatic events, such as the formation of scar tissue that prevents sliding between the meningeal layers, or between the outer meningeal layer and neighboring tissues, chronicizing the dysfunction [86]. In animal models, we know that mild inflammation is sufficient to reduce the function of the phrenic nerve [87]. Let us remember that the presence of a sub-clinical inflammatory environment can lead to the formation of scars between the different tissues, and a phrenic dysfunction cannot always cause symptoms that can easily be linked to the nerve [88,89]. In the absence of known causes, the phrenic nerve can undergo functional alterations (idiopathic).

Symptoms of phrenic dysfunction are not necessarily linked to dyspnea at rest or on exertion but can present as non-specific neck pain or low back pain, alterations in shoulder mobility, mood alterations, a decrease in the pain threshold, alteration of neuro-coordination, and more [27,89-95].

A phrenic nerve has a remarkable ability to adapt, before reaching dysfunction, if the damaging cause becomes chronic. When the phrenic nerve functions in a physiological context, the synaptic plate innervating the diaphragmatic fibers acts in a predominant mode known as "full-collapse," that is, neurosynaptic transmission (exocytosis and endocytosis) is influenced by the electrical frequency [96]. If the phrenic nerve is experiencing a disturbance in its functions, the nerve terminal may act in a predominant "kiss-and-run" mode, that is, the synaptic plate tries to spare neurosynaptic release resources, so that, in the short period, regardless of the electrical frequency, the diaphragm can function without altering the respiratory rhythm [96].

In the early stages of an inflammatory disorder, and/or in the presence of causes that cause transient hypoxia, the phrenic nerve is induced to improve its electrical function, and/or the contralateral phrenic nerve is stimulated to increase its nerve impulses to the diaphragm. This mechanism is known as the "crossed phrenic phenomenon" and allows the respiratory functional effectiveness to be maintained [97-99].

Non-immediate dysfunction of the phrenic nerve

As written in the previous section of the article, the symptoms that can derive from the phrenic nerve in the presence of a chronic irritant cause do not appear immediately.

Furthermore, some body regions that can become the site of the phrenic symptom are not easily linked in clinical reasoning to a dysfunction of the phrenic nerve. This can be explained, on the one hand, by the anastomoses described, and on the other by the ability of the nerve to transport multiple substances in retrograde (centripetal transport) and anterograde (centrifugal transport) modes, involving anatomical structures that are not always neighboring and not necessarily directly connected.

The nerve is a crossroads of metabolic information, incessantly transporting enzymes, various proteins, chemical transmitters, synaptic vesicles, membrane components, growth factors, microRNAs (miRs), monomeric subunits of neurofilaments, and mitochondria [9,27,100,101]. The transport of this numerous metabolic information requires energy (adenosine triphosphate, ATP), with different transit speeds. From the neural soma to the target tissue or from the synaptic plate to the medullary area, a molecule can travel slowly (1 to 10 mm per day) or quickly (1000 mm per day) [9]. These substances help regulate neural functions and influence surrounding tissues [27]. The axon can transport not only biological substances in a physiological regime, but also viruses, bacteria, and inflammatory substances, influencing the areas through which the nerve passes and perpetuating and enlarging a pathological environment [102-105].

Phrenic anastomoses can be a vehicle for non-physiological information. A phrenic dysfunction, and corresponding disorder of the diaphragm muscle, can cause an alteration of the mechanical behavior of the shoulder with pain, probably negatively involving the functional behavior of the nerves related to the motricity of the scapulohumeral joint [106-108].

A phrenic disorder that impacts diaphragmatic function could lead to problems with pain and visceral dysfunction of the pelvic floor, on the one hand, due to the formation of inadequate abdominal pressures, and on the other, due to neurological relationships that can negatively affect the respiratory centers (bulb, pons, and midbrain) [109].

Phrenic dysfunction could cause functional alterations of the lingual complex and related symptoms, such as dysphagia and sleep apnea, probably due to neurological relationships with the cranial nerves that innervate the tongue area, as previously described [27,89,91,110,111]. A dysfunction of the phrenic nerve could increase blood pressure, thanks to the connection with the sympathetic system, increasing sympathetic neural outflow [16]. The dysfunctional phrenic nerve could be responsible for chronic dentoalveolar pain, regardless of previous treatments of the oral cavity or the presence/absence of the tooth. The motivation may lie in phrenic afferents. The irritated phrenic nerve sends afferents via the spinotrigeminal nerve, which negatively affects the trigeminal nerve up to the alveolar nerve (mandibular or maxillary), creating an inflammatory and painful environment [27].

Working the phrenic nerve means not only improving its local function but also being able to obtain a systemic benefit thanks to the associated neuro-anatomical relationships.

Osteopathic manual approach to the phrenic nerve

We have no data in the literature on the local or systemic effect after a manual osteopathic medicine treatment on the most superficial area of the phrenic nerve or peripheral nerves, despite it being feasible to use palpation on the same anatomical regions, and the scientific assumptions that would support such a clinical practice. The approach we propose is, therefore, a theory based on clinical experience and the rationale that we can extrapolate from the literature. Let us remember that the meaning of evidence-based medicine (EBM), a philosophy born between 1950 and 1960, is “The practice of evidence-based medicine means integrating individual clinical expertise with the best available external clinical evidence from systematic research. By individual clinical expertise, we mean the proficiency and judgment that individual clinicians acquire through clinical experience and clinical practice…..and in the more thoughtful identification and compassionate use of individual patients' predicaments, rights, and preferences in making clinical decisions about their care” [112].

In good clinical practice, the experience of the clinician is equally important. We hope that the article will be an incentive for further experimental investigations using the technique we are going to illustrate.

We must consider osteopathic manual work on the nerve as a gentle fascial treatment: “The fascia is the philosophy of the body, meaning each body region is connected to another, whereas osteopathy is the philosophy of medicine: the entire human body must work in harmony” [72].

The nerve is among the most elastic and resistant tissues: you should not be afraid of causing damage if you press. You should never be in a hurry to find a landmark and the nerve itself; the nerve can manifest itself under our fingers as a thin thread or cord. Once you reach the nerve, there is no need to press further: you can imagine a tube with a finger on it. Touching the tube is part of the objective.

The most superficial portion of the phrenic nerve, which we can palpate and treat, is lateral to the clavicular muscle area of the sternocleidomastoid muscle (SCOM). To facilitate the landmarks, we can passively rotate the patient's neck toward the opposite side to the palpation of the nerve; if we have to manually work the right phrenic, we can rotate the person's head (who is in the supine position) to the left. This will highlight the clavicular area of the SCOM.

**Figure 1:**
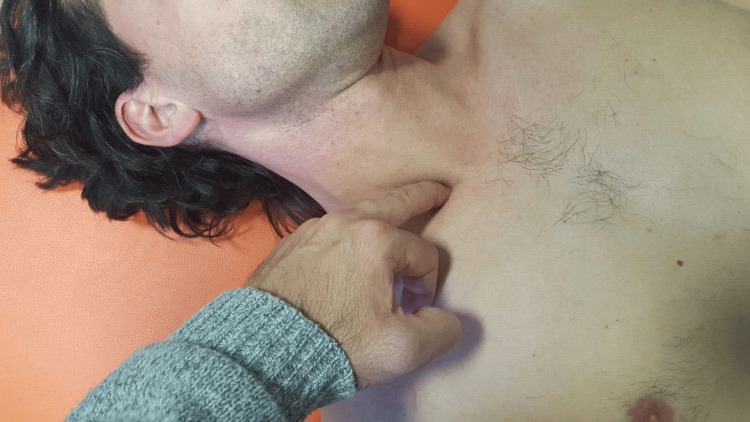
The image shows the anatomical area where the osteopathic clinician places the finger, with the patient in the supine position. The pressure must always be acceptable to the patient. Image Credit: Bruno Bordoni.

The identification of the phrenic nerve, in cases where it is difficult to palpate the nerve at the root of the neck, can also be carried out with ultrasonography, where the nerve's hyperechoic rim is highlighted on the right and the left [113]. Once the nerve has been identified (via palpation or with ultrasound), a finger is placed, and the listening technique described previously is carried out (Figure 1). By palpation, the clinician may discern different parameters, such as the tone heard, or possible movements of the nerve caused by breathing. Such parameters may change to a positive mode (the tone decreases, or the movements you perceive are homogeneous). At that point, the manual approach is complete. The time needed to achieve this result will depend on the patient's tissue.

The osteopathic clinician can decide, based on the clinical history collected or on the general palpation carried out, to target the phrenic nerve only on one side of the neck or bilaterally in succession. The pressure must always be adapted to the patient. From clinical experience, if the phrenic nerve is in dysfunction, the palpated area of the phrenic nerve will be slightly painful; although we must not exclude that the same neighboring tissues may be painful due to other pathognomonic causes. Depending on the clinical picture and the patient's symptomatic response, the approach can be repeated in other sessions, or just one may suffice to help the patient. Again, according to the osteopath's assessment, the proposed manual approach can be the focus of the treatment, it can be the preparation for another type of treatment and for another body area, or as a technique to conclude the osteopathic session. For readers who wish to delve deeper into the topic of manual treatment of peripheral nerves, we recommend reading the English version of Barral and Croibier's book [114]. There are many manual approaches that the osteopathic clinician could use, but the gentle approach that we illustrate in the article is chosen because it does not cause any side effects, respects the palpated tissue, and does not increase the patient's pain. The therapeutic effect will depend on the experience of the operator and/or the weight of the underlying dysfunction. The choice of how to set up the treatment will depend on the evaluation ability of the individual operator. Palpation in osteopathic medicine is the basis for diagnosis and treatment, moving from the skin to the desired tissue depth. The osteopath after long school training is able to use palpation as a fundamental tool in the clinical setting; palpation guides the osteopath [115]. Palpation and subsequent treatment of the peripheral nerve is a feasible strategy.

## Conclusions

The article reviewed the anatomical path of the phrenic nerve and its anastomoses, with the most up-to-date information. The text briefly reviewed possible nerve dysfunctions, as well as the nerve's ability to transport numerous biological substances in anterograde and retrograde modes. We have tried to formulate a scientific rationale to support an osteopathic manual treatment on the most superficial portion of the phrenic nerve, thus laying the foundations for further experimental studies on the feasibility of the proposal presented in the article. The theoretical proposal on palpation and osteopathic treatment of the phrenic nerve is based on the authors' almost 30 years of clinical experience, and as mentioned in the body of the text, the operator's clinical experience is an integral part of the vision of evidence-based medicine.
